# Alternative microbial-based functional ingredient source for lycopene, beta-carotene, and polyunsaturated fatty acids

**DOI:** 10.1016/j.heliyon.2023.e13828

**Published:** 2023-02-17

**Authors:** Chewapat Saejung, Khomsorn Lomthaisong, Prawphan Kotthale

**Affiliations:** a Department of Microbiology, Faculty of Science, Khon Kaen University, Khon Kaen, 40002, Thailand; b Department of Biochemistry, Faculty of Science, Khon Kaen University, Khon Kaen, 40002, Thailand

**Keywords:** PUFAs, Lycopene, Beta-carotene, Microbial lipids, Soybean oil

## Abstract

The acquisition of carotenoids and polyunsaturated fatty acids (PUFAs) from plants and animals for use as functional ingredients raises concerns regarding productivity and cost; utilization of microorganisms as alternative sources is an option. We proposed to evaluate the production of carotenoids and PUFAs by *Rhodopseudomonas faecalis* PA2 using different vegetable oils (rice bran oil, palm oil, coconut oil, and soybean oil) as carbon source, different concentrations of yeast extract as nitrogen source at different cultivation time to ensure the best production. Cultivation with soybean oil as source of carbon led to the most significant changes in the fatty acid profile. Compared to the initial condition, the strain cultivated in the optimal conditions (4% soybean oil, 0.35% yeast extract, and 14 days of incubation) showed an increase in μ_max_, biomass, carotenoid productivity, and microbial lipids by 102.5%, 52.7%, 33.82%, and 34.78%, respectively. The unsaturated fatty acids content was raised with additional types of PUFAs; omega-3 [alpha-linolenic acid and eicosapentaenoic acid] and omega-6 [linoleic acid and eicosatrienoic acid] fatty acids were identified. The results of ultra high-performance liquid chromatography-electrospray ionization-quadrupole time of flight-mass spectrometry (UHPLC-ESI-QTOF-MS/MS) indicated the molecular formula and mass of bacterial metabolites were identical to those of lycopene and beta-carotene. The untargeted metabolomics revealed functional lipids and several physiologically bioactive compounds. The outcome provides scientific reference regarding carotenoids, PUFAs, and useful metabolites that have not yet been reported in the species *Rhodopseudomonas faecalis* for further use as a microbial-based functional ingredient.

## Highlights

• Additional types of PUFAs are found in the proposed condition.
• Beta-carotene and EPA are first reported in *Rhodopseudomonas faecalis.*
• Functional lipids and physiologically bioactive compounds were observed.
• *R. faecalis* PA2 might be used as a new microbial-based functional ingredient.
• The optimal conditions enhance μ_max_, biomass, carotenoids, and microbial lipids.

## 1 Introduction

Increasing concern for health and well-being has increased the inclusion of functional ingredients in dietary supplements, nutraceuticals, and health products. Carotenoids and polyunsaturated fatty acids (PUFAs) are examples of high-value compounds supplemented in many products. Carotenoids, natural biomolecules produced by plants, algae, and some bacteria, have been shown to have provitamin A activities and strong antioxidant potential, allowing them to fight cancer, age-related macular degeneration, photooxidative damage, and boost immunological response [1]. Carotenoids have received interest in the nutraceuticals and food industries, with a market worth of around 1.21 billion USD [2,3]. Functional lipids are compounds involved in a broad spectrum of metabolic conditions. Long-chain PUFAs in *n*-3 and *n*-6 series (omega-3 and omega-6 fatty acids) have been discovered as the essential fatty acids in mammals because of their specific biofunctions as precursors for eicosanoids that modulate pulmonary function [4], structural components of membranes, and inflammatory responses [5]. There is plenty of data to show that PUFAs can help avoid a variety of chronic diseases [6]. Alpha-linolenic acid (18:3, *n*-3; ALA), eicosapentaenoic acid (20:5, *n*-3; EPA), docosahexaenoic acid (22:6, *n*-3; DHA), linoleic acid (18:2, *n*-6; LA), and arachidonic acid (20:4, *n*-6) are all examples of essential fatty acids. Carotenoids and PUFAs cannot be biosynthesized by human body, making them the crucial functional ingredients in several products.

There are many carotenoid-based products on the market, as well as dietary supplements with PUFAs [7,8]. Carotenoids and PUFAs derived from plants and animals raise concerns not only their productivity but also production cost. PUFAs are found in high-price food such as chia seeds, fish oils, and marine fish in the families *Scombridae*, *Clupeidae*, and *Salmonidae* [9]. Plant carotenoids require agricultural land, pesticides, growing season, and harvesting time. Therefore, carotenoids and PUFAs acquire from these sources as the functional ingredients are expensive. At present, the use of microbial biomass as a functional ingredient is a viable option to provide key nutrients at a cheaper cost with a higher yield [10]. As a result, microorganisms are increasingly used in functional food, functional ingredients, and nutraceuticals businesses. Carotenoids and PUFAs have been acquired from a variety of bacteria, fungi, and microalgae for use as active ingredients in industries. Beta-carotene from *Sphingomonas* sp. and canthaxanthin from *Paracoccus carotinifaciens* are examples of bacterial carotenoids used in food colorants [3] while lycopene from *Rhodospirillum rubrum* and spheroidenone from *Rhodobacter sphaeroides* have been shown to have anti-cancer and anti-inflammatory properties in health products [11]. Nutritional supplements containing beta-carotene and astaxanthin derived from the algae *Dunaliella salina* and *Haematococcus pluvialis* are also reported [12]. *Mortierella alpina*, *Mortierella alliacea* [6], and *Rhodotorula mucilaginosa* are among the fungal producers of omega-3 and omega-6 fatty acids [13]. A heterotrophic unicellular marine thraustochytrid *Aurantiochytrium* sp. [14]. and a microalga *Crypthecodinium cohnii* [15] have been used as sources of DHA and squalene. Several significant metabolites from microorganisms are currently investigated to explore their application and utilization as functional ingredients.

The anoxygenic photosynthetic bacteria are excellent producers of carotenoids and PUFAs. Because of their several modes of metabolism, these bacteria have been widely used in waste treatment. They are not pathogens but they do contain several types of useful compounds such as coenzyme Q_10_, 5-aminolevulinic acid, carotenoids, bacteriochlorin, and polyhydroxyalkanoates [16]. They have membrane lipids and phospholipids which are not typically found in general bacteria, such as phosphatidylcholine, sulfoquinovosyldiacylglycerol, betaine lipids, and ornithine lipids [17]. Although their prominent characteristics have been reported to be used as single-cell protein (SCP) and feed [18], they have not yet received the attention they deserve, and the information about utilization of these bacteria as functional ingredients is scarce. Lycogen™, a carotenoid product acquired from *Rhodobacter sphaeroides* WL-APD911, is the only product obtained from anoxygenic photosynthetic bacteria utilized in mammals that shows anti-inflammatory, anti-oxidative, and glucose homeostasis effects [11,19].

The anoxygenic photosynthetic bacterium *Rhodopseudomonas faecalis* PA2 contains several nutrients [20] and high protein content containing all essential amino acids [21] although it was cultivated on waste substrates. Aquatic animals fed *R. faecalis* PA2 showed superior performances and survival in comparison with animals fed the alga *Chlorella vulgaris*, the yeast *Saccharomyces cerevisiae*, the cyanobacterium *Spirulina* sp., and the other species of anoxygenic photosynthetic bacteria [22,23]. This indicates it could be a potential candidate for application in functional ingredient industries and the investigation of additional useful metabolites of this strain has drawn attention.

To produce the microbial-based functional ingredients, carbon source for microbial growth is crucial and the expense of carbon source has to be factored in. Organic acids, such as malic acid and succinic acid, are the essential carbon for anoxygenic photosynthetic bacteria but they are the expensive feedstock. On the other hand, vegetable oils are much cheaper; the catabolism of oil components produces organic acids as intermediates that can be used for the growth of anoxygenic photosynthetic bacteria [24]. Hence, the objectives of this study were to identify lycopene, beta-carotene, and PUFAs in the anoxygenic photosynthetic bacterium *R. faecalis* PA2 in the presence of vegetable oils and to evaluate the metabolite profiling of this strain. In this study, a Liquid Chromatography-Mass Spectrometry (LC-MS)-based metabolomic approach was used to investigate the metabolic composition, aiming to reveal the interesting metabolites in this strain. To the best of our knowledge, this is the first study that used metabolomics to quantify the useful metabolites and to observe the metabolites which have not been reported in the anoxygenic photosynthetic bacteria.

## 2 Materials and methods

### 2.1 Effects of vegetable oils as carbon sources on biomass, carotenoids, microbial lipids, and fatty acid composition

The photosynthetic bacterium *R. faecalis* PA2 was employed which is safely deposited at Thailand Bioresource Research Center (TBRC 5694) for research and commercial purposes. The cultivation of this strain and inoculum preparation were carried out in glutamate-malate medium and exposed to light intensity at 4000 lux under anoxygenic conditions [25]. The basal medium (BM) supplemented with 1% vegetable oil as a carbon source (rice bran oil, palm oil, coconut oil, or soybean oil) was used as the tested medium and adjusted pH to 6.8; inoculum volume was 10%. Incubation was carried out at 30 ± 2 °C under light-anoxygenic conditions. The experiments were conducted in six replicates. Biomass, carotenoid, and microbial lipid concentrations were investigated at intervals of 48 h. Bacterial cells were separated from the culture broth by centrifugation at 6000 rpm 4 °C for 10 min at the end of the experiment (Himac CR20B2, Hitachi, Tokyo, Japan). The supernatant was discarded; the cell pellets were washed with 0.85% sterile NaCl and then freeze-dried using a freeze dryer (Freezone 2.5 L; LABCONCO, KC, USA). The fatty acid composition of the freeze-dried biomass was determined following AOAC [26] method 996.06. Briefly, the Shimadzu Nexis GC-2030 equipped with split injector port, flame ionization detector (FID), and AOC-20i + s autosampler was used. The fatty acid methyl ester (FAME) mix was analyzed according to the AOAC method 996.06 which required the use of helium carrier gas (constant linear velocity 18 cm/s). The column was Rt-2560 100 m × 0.25 mm ID × 0.20 μm film thickness. The GC parameters included inlet (1 μL split injection; 225 °C; split ratio 200:1) and flame ionization detector (285 °C; H_2_ 32 mL/min; air 200 mL/min; make-up (N_2_) 24 mL/min). The oven temperature was 100 °C (4 min hold); 3 °C/min to 240 °C (15 min hold). The FAME mix was purchased from Restek (PA, USA).

### 2.2 Optimization of cultural condition

Since carbon content, nitrogen (yeast extract) content, and incubation period play the significant roles in boosting bacterial growth and essential metabolites, these three parameters were investigated. The optimization was carried out by one-variable at a time. The optimal vegetable oil was used as a carbon source in BM. The vegetable oil content (1%, 2%, 4%, 6%, 8%, and 10% (w/v)) was supplemented in BM and adjusted pH to 6.8. The 10% inoculum was included. The experiment was set for 10 days at 30 ± 2 °C under light-anoxygenic conditions. The biomass, carotenoid, and microbial lipid concentrations were investigated at intervals of 48 h. Six duplicates of each experiment were carried out. For the optimization of yeast extract content, the contents of 0.05%, 0.10%, 0.15%, 0.20%, 0.25%, 0.30%, 0.35%, 0.40%, 0.80%, and 1.60% were optimized. The incubation period of 6, 8, 10, 12, and 14 days were investigated. The incubation conditions were carried out as stated.

### 2.3 Bacterial cultivation under the optimal conditions and determination of fat and fatty acid composition

*Rhodopseudomonasfaecalis* PA2 was grown in a photo-bioreactor with 10% inoculum under optimal conditions. Nitrogen gas was flushed into the reactor to create an anoxygenic condition. Illumination (4000 lux) was provided throughout the experiment. Bacterial cells were freeze-dried and used to determine total fat and fatty acid composition by the hydrolytic extraction gas chromatographic technique.

### 2.4 Cell extraction for metabolite measurement

The wet cells were used, and 50 mg of the sample (five replicates) was dissolved with 1 mL reconstitution buffer (water: acetonitrile = 1:1). The mixture was sonicated for 15 min three times (Ultrasonic Cleaner GT SONIC-D2, GT SONIC, Shenzhen, China) and centrifuged at 15 000 rpm at 4 °C for 15 min twice (D3024R High Speed Refrigerated Micro Centrifuge, DLAB, DLAB Scientific, Beijing, China). The supernatant was transferred to the high-performance liquid chromatography (HPLC) glass vial for LC-MS data acquisition.

### 2.5 Determination of carotenoids and untargeted profiling of metabolites using Ultra High-Performance Liquid Chromatography - Electrospray Ionization - Quadrupole Time of Flight - Mass Spectrometry (UHPLC-ESI-QTOF-MS/MS) analysis

Standard lycopene and beta-carotene were used for quantification of the detected carotenoids using the targeted metabolite analysis. The extracted samples were analyzed on reverse-phase liquid chromatography. The separation was performed using UHPLC-ESI-QTOF-MS/MS (Bruker Daltonics, Bremen, Germany). Bruker intensity solo HPLC C18 2.1 × 100 mm, 2 μm column was used (Bruker Daltonics, Bremen, Germany). The column temperature and autosampler temperature were maintained at 40 °C and 4 °C, respectively. The mobile phase consisted of eluent A (100% water and 0.1% formic acid (FA)) and eluent B (100% acetonitrile and 0.1% FA). The flow rate was 0.35 mL/min; the gradient elution was set as follow: 99-5% A (0.0–4.0 min, 0.25 mL/min), 5% A (4.0–8.1 min, 0.25 mL/min), and 99% A (8.1–11.0 min, 0.25 mL/min). Injection volume was 2 μL applied for positive ionization polarity mode. The mass spectrometry was performed using the broadband collision‐induced dissociation (bbCID) method by a compact ESI-Q-TOF system (Bruker Daltonics, Bremen, Germany). Sodium formate solution (2 mM sodium hydroxide, 0.1% FA, 50% isopropanol) was injected as an external calibrant with a flow rate of 0.5 μL/min. The condition in positive ionization polarity mode consisted of 50–1300 *m*/*z* mass range, 35 V cone voltage, 4000 V capillary voltage, 220 °C source temperature, 220 °C desolvation temperature, and 8 L/min desolvation gas flow.

For the untargeted metabolite profiling analysis, the flow rate was adjusted to 0.35 mL/min; the gradient elution was set as follows: 99% A (0.0–2.0 min, 0.25 mL/min), 1% A (2.0–20.0 min, 0.25 mL/min), 99% A (20.1–28.3 min, 0.35 mL/min), and 99% A (28.5–30.0 min, 0.25 mL/min). Injection volume was 2 μL applied for positive and negative ionization polarity modes. The conditions in positive ionization polarity mode are 50–1300 *m*/*z* mass range, 35 V cone voltage, 4000 V capillary voltage, 220 °C source temperature, 220 °C desolvation temperature, and 8 L/min desolvation gas flow. The conditions in negative ionization polarity mode are 50–1300 *m*/*z* mass range, 31 V cone voltage, 4500 V capillary voltage, 220 °C source temperature, 220 °C desolvation temperature, and 8 L/min desolvation gas flow.

### 2.6 Metabolite identification and annotation

The data was imported to the MetaboScape software for metabolite identification. The assessment of metabolites was compared with the public database: METLIN, Human Metabolome Database (HMDB), Bruker MetaboBASE, and LipidBlast database. Level of assignment (LoA) of the metabolites include 1) accurate mass matched to the database, 2) accurate mass matched to database and tandem MS spectrum matched to in silico fragmentation pattern, 3) tandem MS spectrum matched to database or literature, 4) retention time and the molecular mass matched to standard compound, and 5) MS/MS spectrum matched standard compound.

### 2.7 Analytical procedures

Bacterial biomass and carotenoids were analyzed according to Saejung and Chanthakhot [25]. Carotenoids were extracted by immersing the cell pellets in methanol-acetone (2:3 v/v) solution overnight until the colorless cells were obtained. The pigment extract was read at 480 and 770 nm using a Genesys 20 spectrophotometer (Thermo Scientific, Waltham, MA, USA). Microbial lipids were extracted from cells by centrifugation of the culture broth at 9000 rpm 4 °C for 15 min. The pellets were resuspended in distilled water after being washed twice with 0.9% NaCl. The pellets were boiled for 10 min in 1 N NaOH, and the cell debris was discarded [27]. The supernatant was used to examine microbial lipids by saponification with 1.5 M KOH in 80% ethanol following Kwon and Rhee [28].

### 2.8 Statistical analyses

The data were presented as mean ± standard deviation (SD). The significant differences between means were calculated by one-way analysis of variance (ANOVA). The Duncan's multiple range test was used to compare the means at a significance level of *p* ≤ 0.05. The software IBM SPSS Statistics 28.0.0.0 (IBM Corp., Armonk, NY, USA) was used for statistical analyses.

## 3 Results

### 3.1 Effects of vegetable oils as carbon sources on biomass, carotenoids, microbial lipids, and fatty acid composition

**Fig. 1:**
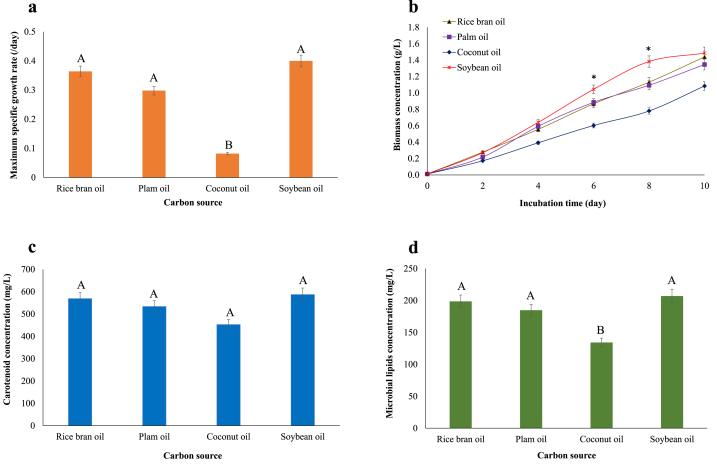
Growth and microbial substances of *Rhodopseudomonas faecalis* PA2 cultivated in basal medium containing different vegetable oils as carbon sources. (a) μ_max_, (b) biomass concentration, (c) carotenoid concentration, and (d) microbial lipid concentration. Different superscript letters in each bar indicate significant differences among treatments (*p* ≤ 0.05). ∗ denotes that the value was significant difference.

All the tested vegetable oils could be used as sole carbon‐based nutrients. This phenomenon is supported by Fig. 1a-d, which depicts the growth of *R. faecalis* PA2 and the generation of some metabolites in the presence of vegetable oils. The use of coconut oil as a carbon source resulted in the lowest maximum specific growth rate (μ_max_) (0.082 ± 0.005/day), carotenoid concentration (452.58 ± 8.56 mg/L), and microbial lipid concentration (134.28 ± 7.66 mg/L). Soybean oil, on the other hand, showed the highest values. The biomass of *R. faecalis* PA2 fed coconut oil had the highest saturated fatty acid content (Table 1). However, cultivation with soybean oil showed the predominant fatty acid composition because the biomass of *R. faecalis* PA2 contained both omega-3 fatty acid (ALA) and omega-6 fatty acids (LA and eicosatrienoic acid [or dihomo-gamma-linolenic acid (DGLA]). Therefore, soybean oil was employed in the following experiments due to the composition of unsaturated fatty acids.

**Table 1:** Total fat and fatty acid composition of *Rhodopseudomonas faecalis* PA2 cultivated in basal medium containing different vegetable oils as carbon sources.

Fatty acids	Content (g/100 g)			
	Rice bran oil	Palm oil	Coconut oil	Soybean oil
**Saturated fatty acids**	7.491^a^	6.742^a^	8.295^a^	7.098^a^
Myristic acid (14:0)	–^a^	0.249 **±** 0.12^a^	0.985 **±** 0.22^b^	0.212 **±** 0.01^a^
Palmitic acid (16:0)	6.384 **±** 1.22^a^	5.467 **±** 0.85^a^	4.882 **±** 1.82^a^	5.363 **±** 0.78^a^
Heptadecanoic acid (17:0)	-^a^	0.075 **±** 0.01^b^	0.053 **±** 0.02^b^	0.102 **±** 0.22^b^
Stearic acid (18:0)	1.107 **±** 0.11^a^	0.951 **±** 0.41^a^	0.918 **±** 0.44^a^	1.421 **±** 0.75^a^
Caprylic acid (8:0)	-^a^	-^a^	0.121 **±** 0.03^b^	-^a^
Capric acid (10:0)	-^a^	-^a^	0.134 **±** 0.04^b^	-^a^
Lauric acid (12:0)	-^a^	-^a^	1.202 **±** 0^b^	-^a^
**Unsaturated fatty acids**	**5.619**^a^	**4.288**^a^	**2.186**^b^	**4.963**^**a**^
*cis*-10-Heptadecenoic acid (17:1, *n*-10)	-^a^	-^a^	0.048 ± 0^b^	-^a^
Palmitoleic acid (16:1, *n*-7)	1.004 **±** 1.00^a^	0.583 **±** 0.21^b^	0.732 **±** 0.51^ab^	0.726 **±** 0.41^ab^
*cis*-9-Oleic acid (18:1*, n*-9)	2.893 **±** 1.75^a^	2.612 **±** 1.22^a^	0.453 **±** 0.05^b^	2.123 **±** 0.71^ab^
*cis*-9,12-Linoleic acid (18:2, *n-*6)	1.722 **±** 0.71^a^	0.518 **±** 0.45^b^	0.113 **±** 0.45^b^	1.440 **±** 0.15^ab^
alpha-Linolenic acid (18:3, *n*-3)	-^a^	-^a^	-^a^	0.081 **±** 0.05^b^
*cis*-8,11,14-Eicosatrienoic acid (20:3, *n*-6)	-^a^	0.575 **±** 0.15^b^	0.840 **±** 0.11^b^	0.593 **±** 0.21^b^
**Total fat**	**13.11**^a^	**11.03**^a^	**10.48**^a^	**12.06**^a^

### 3.2 Optimization of soybean oil contents

**Fig. 2:**
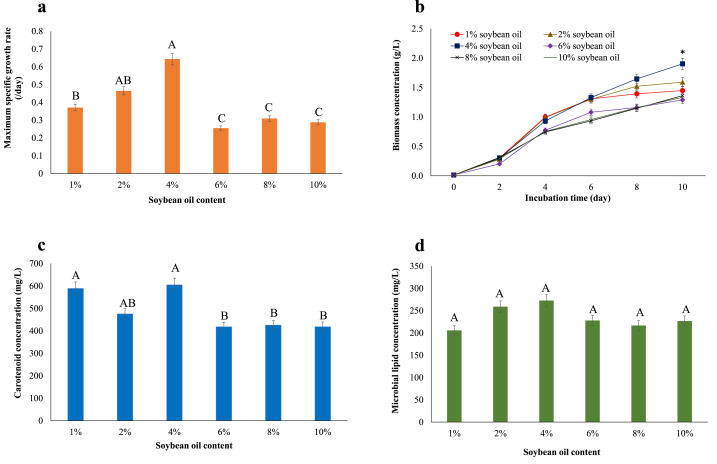
Growth and microbial substances of *Rhodopseudomonas faecalis* PA2 cultivated in basal medium containing different soybean oil contents. (a) μ_max_, (b) biomass concentration, (c) carotenoid concentration, and (d) microbial lipid concentration. Different superscript letters in each bar indicate significant differences among treatments (*p* ≤ 0.05). ∗ denotes that the value was significant difference.

As shown in Fig. 2a, as the content of soybean oil increased, μ_max_ increased, with the maximum level of 0.64 ± 0.01/day, occurring at 4%, above which the μ_max_ decreased. Fig. 2b-d shows that supplementing with 4% soybean oil resulted in the highest biomass, carotenoid, and microbial lipid concentrations; however, there was no statistical difference of microbial lipids among the treatments. Table 2 indicates the μ_max_ at 4% soybean oil was enhanced by 60% when compared to the initial condition (1% soybean oil).

**Table 2:** Comparison of μ_max_, biomass concentration, carotenoid productivity, and microbial lipid concentration of *Rhodopseudomonas faecalis* PA2 grown in each optimization study and initial condition.

Culture condition	μ_max_ (/day)	Biomass concentration (g/L)	Carotenoid productivity (mg/L/day)	Microbial lipid concentration (mg/L)
Initial condition^a^	0.40 ± 0.01	1.48 ± 0.11	587 ± 11.12	207.01 ± 17.05
Optimal soybean oil content (4% soybean oil)	0.64 ± 0.51	1.90 ± 0.44	604.61 ± 25.22	272.68 ± 1.59
% increase compared to initial condition	+60	+28.38	+3	+31.72
Optimal yeast extract content (0.35% yeast extract)	0.94 ± 0.23	2.57 ± 1.01	678.52 ± 24.02	296.46 ± 33.10
% increase compared to initial condition	+135	+73.65	+15.59	+43.21
Optimal incubation time (14 days)	0.81 ± 0.52	2.26 ± 0.99	785.55 ± 14.78	279 ± 20.65
% increase compared to initial condition	+102.50	+52.70	+33.82	+34.78

a 1% soybean oil, 0.2% yeast extract, and 10 days of incubation.

### 3.3 Optimization of yeast extract content

**Fig. 3:**
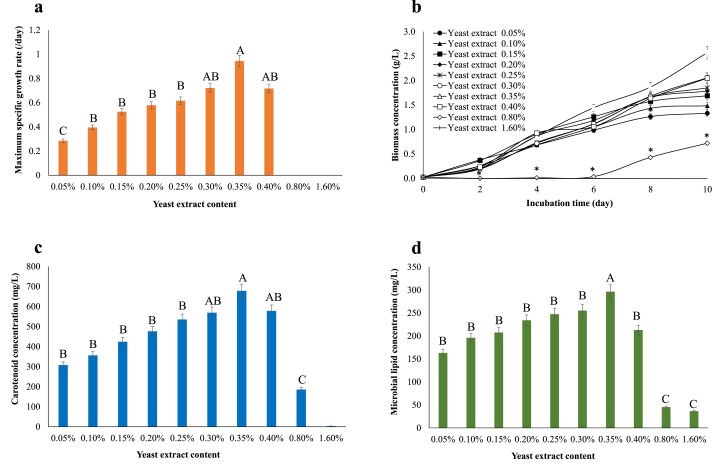
Growth and microbial substances of *Rhodopseudomonas faecalis* PA2 cultivated in basal medium containing different yeast extract contents. (a) μ_max_, (b) biomass concentration, (c) carotenoid concentration, and (d) microbial lipid concentration. Different superscript letters in each bar indicate significant differences among treatments (*p* ≤ 0.05). ∗ denotes that the value was significant difference.

Yeast extract is the most effective nitrogen source for *R. faecalis* PA2 [29], thus, the optimal content should be investigated. The μ_max_ and biomass concentration varied depending on yeast extract content (Fig. 3a and b). The highest μ_max_ and biomass concentration were found at 0.35% yeast extract, with the increase by 135% and 73.65%, respectively (Table 2). Carotenoid synthesis is reduced at lower C/N ratios (0.80%–1.60% yeast extract) (Fig. 3c). The concentration of microbial lipids was dramatically reduced when yeast extract content was greater than 0.35% because of the excessive nitrogen level (Fig. 3d).

### 3.4 Optimization of the incubation period

**Fig. 4:**
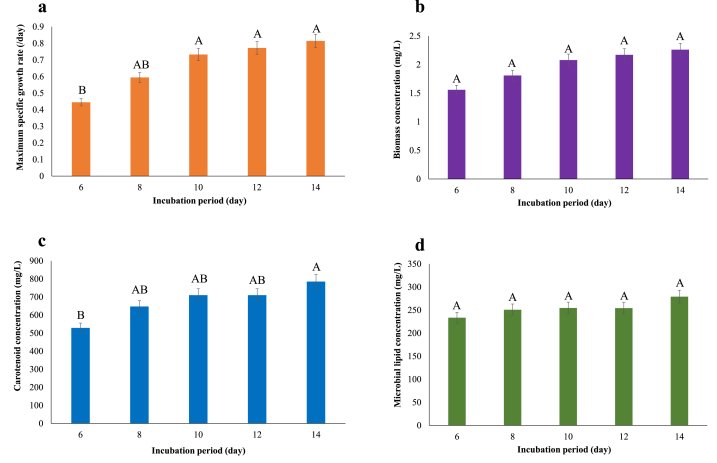
Growth and microbial substances of *Rhodopseudomonas faecalis* PA2 cultivated in different incubation period. (a) μ_max_, (b) biomass concentration, (c) carotenoid concentration, and (d) microbial lipid concentration. Different superscript letters in each bar indicate significant differences among treatments (*p* ≤ 0.05).

The μ_max_, biomass concentration, carotenoids, and microbial lipid production increased with increasing incubation period, as indicated in Fig. 4a-d, with the maximum at 14 days. In comparison to the initial condition, carotenoids were increase by 33.82% (Table 2). A long incubation period boosted carotenoid production because they are produced during stationary phase. An increase in carotenoids was found after 12 days of incubation (Fig. 4C).

### 3.5 Determination of fatty acid composition

The kinetic characteristics of *R. faecalis* PA2 acquired from a photo-bioreactor are presented in Table S1. Table 3 indicates the total fat and fatty acid composition of *R. faecalis* PA2 cultivated in the optimal conditions compared with the initial condition. The proposed conditions (4% soybean oil, 0.35% yeast extract, and 14 days of incubation) could enhance the content of unsaturated fatty acid significantly compared with the initial condition. The unsaturated fatty acids *cis*-10-pentadecenoic acid (15:1, *n*-10), *cis*-10-heptadecenoic acid (17:1, n-10), and EPA were present in the optimal conditions, whereas they were not found in the initial condition (Table 3). This study revealed that ALA, EPA, LA, and DGLA were found in the biomass of *R. faecalis* PA2 grown in the proposed conditions. To our knowledge, EPA was first reported in this species. Therefore, using soybean oil as carbon source along with the conditions described in this study might provide the important PUFAs in *R. faecalis* PA2.

**Table 3:** Total fat and fatty acid composition of *Rhodopseudomonas faecalis* PA2 cultivated in the optimal conditions compared with the initial condition.

Fatty acids	Content (g/100 g)	
	Optimal condition^a^	Initial condition^b^
**Total fat**	**12.25**^a^	**12.06**^a^
**Saturated fatty acids**	**3.215**^**a**^	**7.098**^**b**^
Lauric acid (12:0)	0.140 ± 0.15^a^	-^b^
Myristic acid (14:0)	0.212 ± 0.01^a^	0.212 ± 0.02^a^
Pentadecanoic acid (15:0)	0.025 ± 0^a^	-^ab^
Palmitic acid (16:0)	2.294 ± 1.00^a^	5.363 ± 0.24^b^
Heptadecanoic acid (17:0)	0.067 ± 0.02^a^	0.102 ± 0.01^a^
Stearic acid (18:0)	0.477 ± 0.01^a^	1.421 ± 0^b^
Arachidic acid (20:0)	–	–
**Unsaturated fatty acids**	**9.034**^**a**^	**4.963**^**b**^
*cis*-10-Pentadecenoic acid (15:1, *n*-10)	0.020 ± 0^a^	**-**^ab^
Palmitoleic acid (16:1, *n*-7)	0.407 ± 0.05^a^	0.726 ± 0.71^a^
*cis*-10-Heptadecenoic acid (17:1, *n*-10)	0.053 ± 0.01^a^	- ^ab^
*cis*-9-Oleic acid (18:1*, n*-9)	7.491 ± 1.11^a^	2.123 ± 0.07^b^
*cis*-9,12-Linoleic acid (18:2, *n-*6)^c^	0.084 ± 0.04^a^	1.440 ± 0.23^a^
alpha-Linolenic acid (18:3, *n*-3)^c^	0.042 ± 0^a^	0.081 ± 0.07^a^
*cis*-8,11,14-Eicosatrienoic acid (20:3, *n*-6)^c^	0.231 ± 0.01^a^	0.593 ± 0.12^b^
*cis*-5,8,11,14,17-Eicosapentaenoic acid (20:5, *n*-3)^c^	0.706 ± 0.13^a^	-^b^
Total monounsaturated fatty acids	7.971 ± 1.11^a^	2.849 ± 0.58^b^
Total polyunsaturated fatty acids	1.063 ± 0.12^a^	2.114 ± 0.49^a^
**Unsaturated: Saturated fatty acids ratio**	**2.81**^**a**^	**0.70**^**b**^
**Omega-6: Omega-3 ratio**	**0.42:1**	**25.1:1**

Different superscript letters in each bar indicate significant differences among treatments (*p* ≤ 0.05).

a Cultivation in 4% soybean oil, 0.35% yeast extract, and 14 days of incubation.

b Cultivation in 1% soybean oil, 0.2% yeast extract, and 10 days of incubation.

c Polyunsaturated fatty acids (PUFAs).

### 3.6 Determination of carotenoids and the untargeted profiling of metabolites using UHPLC-ESI-QTOF-MS/MS

**Fig. 5:**
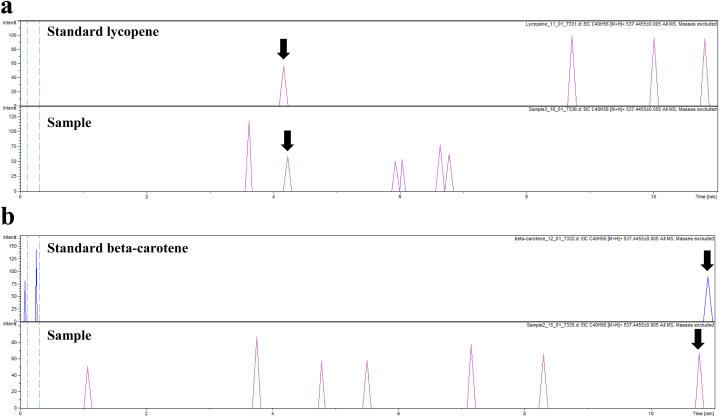
The extracted ion chromatograms (EIC) of metabolites in *Rhodopseudomonas faecalis* PA2 analyzed by using Ultra High-Performance Liquid Chromatography-Electrospray Ionization-Quadrupole Time of Flight-Mass Spectrometry. (a) Lycopene and (b) beta-carotene.

Lycopene and beta-carotene are dietary carotenoids found in fruits and vegetables. They play a vital role in providing health benefits due to their anti-oxidant properties [30]. Therefore, these two carotenoids were quantified using UHPLC-ESI-QTOF-MS/MS-based targeted metabolomics. Fig. 5a and b shows the extracted ion chromatograms (EIC) of the standard carotenoids compared with the samples. The molecular formula and mass of the samples were identical to that of the standard lycopene and beta-carotene. The adduct ions were [M+H]^+^ which indicated that the additional molecule was the proton. The molecular formula, exact mass (excluding the mass of adduct ion), and the concentration of each carotenoid in bacterial cells are summarized in Table 4. Although the mass of lycopene and beta-carotene was identical, the time that they were eluted was different which was used to identify the carotenoid type. The molecular formula and exact mass of the detected samples showed that this strain contained lycopene and beta-carotene. As far as we know, beta-carotene has not been reported in the species *Rhodopseudomonas faecalis*. To the best of our knowledge, this is the first study to report beta-carotene and lycopene in *R. faecalis* verified by the targeted metabolomic analysis.

**Table 4:** Summary of the commercial carotenoids of *Rhodopseudomonas faecalis* PA2 cultivated in the optimal conditions.

Carotenoids	Molecular formula	Mass (Dalton)	Concentration (μg/mL)
Lycopene	C_40_H_56_	536.438	5.55 ± 0.25
Beta-carotene	C_40_H_56_	536.438	7.18 ± 0.77

**Fig. 6:**
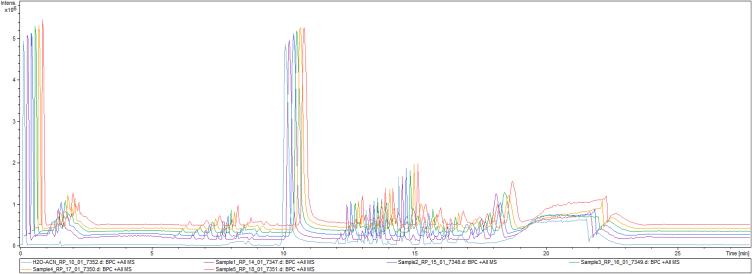
The base peak chromatograms (BPC) of the untargeted metabolite profiling of *Rhodopseudomonas faecalis* PA2 analyzed by using Ultra High-Performance Liquid Chromatography-Electrospray Ionization-Quadrupole Time of Flight-Mass Spectrometry.

The untargeted metabolite profile chromatogram of the samples (five replicates) in positive ionization mode is shown in Fig. 6. The five samples showed identical peaks and there were several metabolites found in this train in the untargeted mode. Each metabolite was identified by comparing with the public database. The identified metabolites are presented in Table 5; the relative concentration of all metabolites was quantified with the external calibrants. Table 6 summarizes the useful metabolites acting as functional ingredients or physiologically bioactive compounds and their benefits. The results also showed several types of phospholipids found in egg yolk, meat, and nuts including lysophosphatidylcholine (LPC), phosphatidylethanolamine (PE), and phosphatidylcholine (PC or lecithin) (Table 6). A microbially associated metabolite, desaminotyrosine, has been found. Buddledin A, (E)-2-octenyl butyrate, and piperonyl acetate were also detected in the cells of *R. faecalis* PA2 cultivated in soybean oil under the optimal conditions.

**Table 5:** The identified metabolites of *Rhodopseudomonas faecalis* PA2 cultivated in the optimal conditions.

Name	Molecular formula	Molecular mass (Da)	Retention time (min)	Level of Assignment^a^	Relative concentration (mM)
(2 R)-2-Hydroxy-2-methylbutanenitrile	C_5_H_9_NO	99.06865	3.33	2	0.05338
(E)-2-Octenyl butyrate	C_12_H_22_O_2_	198.1628	16.99	2	0.173841
1-Dehydro-15alpha-hydroxytestololactone	C_19_H_24_O_4_	316.1657	13.01	2	0.388491
2-Naphthylamine	C_10_H_9_N	143.0737	6.87	2	1.067781
3-amino-2-naphthoic acid	C_11_H_9_NO_2_	187.0639	6.45	2	0.27321
4-Hydroxyproline	C_5_H_9_NO_3_	131.0585	1.32	3	2.492853
Acetyl phosphate	C_2_H_5_O_5_P	139.9878	1.89	2	0.112714
Adenosine diphosphate	C_10_H_15_N_5_O_10_P_2_	427.03	2.19	3	0.061917
ADP-ribose	C_15_H_23_N_5_O_14_P_2_	559.0723	1.99	3	0.029767
Alpha, Beta-trehalose	C_12_H_22_O_11_	342.1168	1.33	3	0.22585
Buddledin A	C_17_H_24_O_3_	276.1727	12.91	2	0.486148
D-Glucose 6-phosphate	C_6_H_13_O_9_P	260.0299	1.37	3	0.109871
D-Glycerate 2-phosphate	C_3_H_7_O_7_P	185.9934	1.85	2	0.221452
Desaminotyrosine	C_9_H_10_O_3_	166.0637	14.4	3	1.773245
Homospermidine	C_8_H_21_N_3_	159.1737	1.01	2	1.388442
l-Leucine	C_6_H_13_NO_2_	131.0949	1.54	3	0.553782
L-Tryptophan	C_11_H_12_N_2_O_2_	204.0895	6.45	3	0.067738
Leu Pro Ile Ile	C_23_H_42_N_4_O_5_	454.3137	9	2	0.003141
LPC 16:1	C_24_H_48_NO_7_P	493.3164	11.65	3	0.037486
LPC 16:2	C_24_H_46_NO_7_P	491.3031	17.11	3	0.137033
LPC 18:1	C_26_H_52_NO_7_P	521.3473	12.22	3	0.098474
LPE 14:0	C_19_H_40_NO_7_P	425.2553	14.76	3	0.220672
LPE 16:0	C_21_H_44_NO_7_P	453.2871	16.63	3	0.181552
LPE 16:0	C_21_H_44_NO_7_P	453.2869	17.41	3	1.834661
LPE 16:1	C_21_H_42_NO_7_P	451.2709	14.82	3	0.64706
LPE 16:1	C_21_H_42_NO_7_P	451.2708	15.2	3	1.041493
LPE 16:1	C_21_H_42_NO_7_P	451.2708	15.3	3	0.634426
LPE 17:1	C_22_H_44_NO_7_P	465.2874	16.25	3	0.289735
LPE 18:1	C_23_H_46_NO_7_P	479.3025	17.03	3	1.065491
LPE 18:1	C_23_H_46_NO_7_P	479.3021	17.98	3	3.751037
LPE 19:2 lyzo	C_24_H_46_NO_7_P	491.3021	17.86	3	0.69955
N,N-Dimethylaniline	C_8_H_11_N	121.0893	6.2	3	0.026896
N-Isopropyl-*p*-toluamide	C_11_H_15_NO	177.1153	1.55	2	0.081274
N-Isopropyl-*p*-toluamide	C_11_H_15_NO	177.1154	5.71	2	0.028937
Nα-Acetyl-l-glutamine	C_7_H_12_N_2_O_4_	188.0803	1.43	3	0.110847
*p*-Tolualdehyde	C_8_H_8_O	120.0579	1.52	2	1.335947
PC 16:0e	C_24_H_50_NO_7_P	495.3334	15.93	3	0.086001
PC 16:1e	C_24_H_48_NO_7_P	493.3184	17.23	3	0.454758
PC 18:1e	C_26_H_52_NO_7_P	521.3481	12.62	3	0.35371
PC 19:2e	C_27_H_52_NO_7_P	533.3478	12.5	3	0.025663
PC 30:1	C_38_H_74_NO_8_P	703.5163	16.17	3	0.1563
PC 32:2	C_40_H_76_NO_8_P	729.5327	16.17	3	0.32134
PC 34:2	C_42_H_80_NO_8_P	757.5619	16.18	3	0.040566
PE 19:2	C_24_H_44_NO_8_P	505.2816	15.41	3	0.684912
PE 19:3	C_24_H_42_NO_8_P	503.2658	12.43	3	1.213597
Phosphoric acid	H_3_O_4_P	97.97706	1.6	2	1.079814
Piperonyl acetate	C_10_H_10_O_4_	194.0559	8.79	2	0.04889
Putrescine	C_4_H_12_N_2_	88.10021	0.99	3	0.094636
Tryptamine	C_10_H_12_N_2_	160.1001	6.9	3	0.140921
Xanthan	C_13_H_10_O	182.0737	13.62	2	1.31766

Leu Pro Ile Ile: short-chain amino acid of Leucine-Proline-Isoleucine-Isoleucine.

LPC: Lysophosphatidylcholine.

LPE: Lysophosphatidylethanolamine.

PC: Phosphatidylcholine.

PE: Phosphatidylethanolamine.

a Metabolite assignment five levels of assignment (LoA): 2 = accurate mass matched to database and tandem MS spectrum matched to in silico fragmentation pattern; 3 = tandem MS spectrum matched to database or literature.

**Table 6:** Summary of the useful metabolites of *Rhodopseudomonas faecalis* PA2 cultivated in the optimal conditions.

Metabolite	Applications	Reference
Lycopene	Nutrient supplement used as antioxidant, anti-cancer, and anti-inflammatory properties.	[11]
Beta-carotene	Nutrient supplement for adult and infant foods which is used as vitamin A precursor.	[55]
Alpha-Linolenic acid (18:3, *n*-3) or ALA	Omega-3 polyunsaturated fatty acid (PUFA) used as nutraceutical against metabolic diseases, inflammatory diseases, and cardiovascular diseases.	[56]
*cis*-5,8,11,14,17-Eicosapentaenoic acid (20:5, *n*-3) or EPA	Omega-3 PUFA used to lowering plasma triglyceride, non-high-density lipoprotein cholesterol (non-HDL-C) levels, and other key lipid/lipoprotein parameters, as well as a broad range of anti-inflammation.	[57]
*cis*-9,12-Linoleic acid (18:2, *n-*6) or LA	Omega-6 PUFA used as supplement to lowering the risk of cardiovascular disease and premature death as well as the active ingredient in moisturizer against skin disorders.	[58,59]
*cis*-8,11,14-Eicosatrienoic acid (20:3, *n*-6) or dihomo-gamma-linolenic acid (DGLA)	Omega-6 PUFA used as a precursor for biosynthesis of biologically active eicosanoids and other metabolites, which has anti-inflammatory, anti-thrombotic, anti-hypertensive, anti-allergic, and anti-proliferation activities.	[[60], [61], [62]]
Phosphatidylcholine and Phosphatidylethanolamine (PE)	Improvement of EPA and DHA levels in brain that can enhance the treatment of depression and neuroinflammatory diseases such as Alzheimer's disease. Reducing atherosclerosis by decreasing plasma very low-density lipoprotein-cholesterol (VLDL-C) and increasing plasma high-density lipoprotein-cholesterol (HDL-C)	[63,64]
Desaminotyrosine	Protection of influenza virus infection through modification of type I interferon signaling and diminution of lung immunology and acting as an anti-inflammatory molecule that contribute to maintain intestinal and systematic immune homeostasis.	[65,66]
Buddledin A	Antifungal action against *Trichophyton rubrum*, *Tricophyton interdigitale*, and *Epidermophyton floccosum*	[67]
Nα-Acetyl-l-glutamine	Prevention of gut damage induced by protein energy malnutrition.	[68]
(E)-2-Octenyl butyrate	Fatty alcohol ester used as flavoring ingredient.	[69]
Piperonyl acetate	Sweet, bitter, and floral tasting compound used as flavoring agent.	[70]

## 4 Discussion

Many studies have been conducted to explore the alternative organisms for the production of carotenoids and PUFAs replacing the production from plants and animals. The use of microorganisms to produce these compounds has increased significantly; yet, few investigations have been undertaken to uncover additional carotenoids and PUFAs producers. In this study, a strategy that used soybean oil as feedstock to produce functional ingredients from beneficial bacterium was established. The strain was able to use vegetable oils by digesting them into glycerol and fatty acids. The glycerol is transformed into dihydroxyacetone phosphate, one of the glycolysis intermediates, and receives energy in the form of ATP through the metabolic process [24]. The fatty acids are metabolized *via* beta-oxidation, which produces either acetyl Co-A or succinyl Co-A depending on the type of fatty acids. Acetyl Co-A is required in the TCA cycle's transition reaction to combine with oxaloacetic acid while succinyl Co-A is one of the intermediates in the TCA cycle, thus, the strain can generate energy by using fatty acids as a carbon source [31]. Rice bran oil, palm oil, coconut oil, and soybean oil have 25%, 49.3%, 83%, and 15% saturated fatty acids, respectively [32,33]. Coconut oil contains the highest content of saturated fatty acids. The saturated fatty acids have higher melting points than unsaturated fatty acids, resulting in requiring more metabolic energy to break down [34]. Photosynthetic bacteria prefer organic acids for growth while coconut oil contains only trace amounts of free fatty acids, thereby influencing the degradation by this strain. Moreover, coconut oil was found to be resistant to microbial degradation in other study [35]. Soybean oil contains 81% unsaturated fatty acids [32], which resulted in facilitating the catabolism by bacteria.

Since multiple intermediates are involved in carotenoid biosynthesis, the important precursor to manufacture these intermediates is acetyl-CoA [36]. As a result, acetyl Co-A acquired from the breakdown of vegetable oils can be employed as a precursor for carotenoid synthesis (Fig. 1c). Moreover, acetyl-CoA is used as a precursor to producing malonyl Co-A *via* carboxylation and then transformed to acetyl-ACP by transacylase for use in lipid biosynthesis. The produced lipids are then transported and stored in bacterial cells [37], hence using vegetable oils as carbon sources aided the buildup of microbial lipids in bacteria. This was in line with prior research reporting the supplementation of phototrophic microorganisms with carbon precursors could increase lipid accumulation [38].

In this study, soybean oil was the only vegetable oil that could boost ALA in the tested strain (Table 1). This was because soybean oil is categorized as an alpha-linolenic acid oil, which contains a significant amount of ALA [39]. ALA, EPA, and DHA are the three important omega-3 fatty acids; DHA and EPA are found in fish and seafood. ALA, on the other hand, can be transformed into EPA and ultimately to DHA [40].

As shown in Fig. 2, μ_max_, carotenoids, and microbial lipid were increased at a certain concentration of soybean oil. This was likely because the high content of carbon source increased the carbon skeleton for the biosynthetic pathway, leading to enhance microbial growth and metabolites [41]. Moreover, the carbon to nitrogen (C/N) ratio of the medium is involved because the greater C/N ratios increase lipid and carotenoid synthesis [42]. Excess carbon supply, on the other hand, caused a decrease in growth rate due to substrate inhibition [43].

The C/N ratio was inversely proportional to the amount of yeast extract present (Fig. 3). When compared to the experiment supplemented with 0.35% yeast extract, the concentrations of yeast extract ranging from 0.05% to 0.30% had a greater C/N ratio. The differences in microbial biomass and respiration reflected these differences. The experiment fed a little amount of yeast extract resulting in the deficiency of nitrogen for biosynthetic pathways at the same amount of carbon. Despite the availability of carbon, the anabolic process comes to a halt. Bacterial growth with a higher C/N ratio is confronted with a surplus of C to N, whereas growth with a lower C/N ratio is confronted with a lack of C to N [44]. As shown in Fig. 4, the longer incubation period resulted in higher biomass, which led to more lipids and carotenoids in bacterial cells. Under the optimal conditions (4% soybean oil, 0.35% yeast extract, and 14 days of incubation), the lipid productivity was 13.86 mg/L/day (Table S1), whereas lipid productivity of *Chlamydomonas reinhardtii*, *Chlorella sorokiniana*, and *Scenedesmus obtusus* XJ-15 were 10.9 mg/L/day, 0.502 mg/L/day, and 0.607 mg/L/day, respectively [[45], [46], [47]]. Previous research reported that carotenoid productivity of *Dunaliella tertiolecta*, *Chlorella vulgaris* UTEX 265, and *Scenedesmus* sp. were 0.86 mg/L/day, 11.98 mg/L/day, and 19.70 mg/L/day, respectively [[48], [49], [50]] while carotenoid productivity of *R. faecalis* PA2 was 45.37 mg/L/day (Table S1). It can be concluded that the lipid productivity and carotenoid productivity of this strain were comparable with the other photosynthetic microorganisms.

The results of this study also prove that changes in the medium composition produce quantitative alteration in fatty acids and carotenoids of anoxygenic photosynthetic bacteria. A previous study showed that the fatty acid composition of bacteria is regulated by the medium composition as well as the age of the cells [51]. This study also calculated the relationships between the ratio of unsaturated to saturated fatty acids (UFA:SFA ratio) because it can be used to evaluate fat utilization. Fat utilization increased with the increase in UFA:SFA ratio; reaching a maximum at UFA:SFA ratio of 4 [52]. Obviously, the strain grown in the optimal condition provided a greater UFA:SFA ratio compared with the initial condition (Table 3). Moreover, previous work also reported the advantage of high UFA:SFA ratio in animal diets in improving meat quality [53]. Omega-6 (LA) can be converted to omega-3 (ALA); thus, the enzymes involved in the metabolism of omega-3 and omega-6 fatty acids are shared and they regulate each other. The balance of omega-6/omega-3 fatty acids in the diet is vital for human nutritional needs. Excessive amount of omega-6 or high omega-6 to omega-3 ratio can cause pathogenesis of diseases [54]. The proportions of omega-6 and omega-3 in the diet can predict the biochemical efficiency, approaching the ratio of 2:1 or 1:1 omega-6/omega-3 fatty acids are the ideal for health. As shown in Table 3, the omega-6/omega-3 fatty acids ratio of *R. faecalis* PA2 cultured in the optimal condition was close to the targeted ratio. *R. faecalis* PA2 contained several metabolites found in foods originating from plants and animals. The detection of functional lipids ALA, EPA, LA, and DGLA in biomass has drawn attention because of their physiological and structural roles in biological systems as shown in Table 6. These metabolites are recognized as high-value compounds supplemented in dietary supplements [56,62], suggesting that the biomass of *R. faecalis* PA2 can be utilized as an alternative source for MUFAs and PUFAs.

The results of UHPLC-ESI-QTOF-MS/MS analysis ensured that the strain and the proposed conditions produced beta-carotene and lycopene. The additional metabolites were detected in cells. The untargeted metabolomics analysis revealed the other functional lipids such as phosphatidylcholine which is a multifunctional phospholipid required for the incorporation of cholesterol in membranes [63]. Our results also showed the presence of desaminotyrosine in *R. faecalis* PA2. According to previous study, this metabolite can protect against influenza virus [65] and maintain systematic immune homeostasis [66]. Nα-acetyl-l-glutamine can be supplemented in sports nutrition's products to help boost exercise endurance and prevent the negative effect of protein energy malnutrition [68]. Buddledin A showed an antifungal effect [67]. (E)-2-octenyl butyrate is used as a flavoring ingredient, whereas piperonyl acetate is found in the green vegetables [69,70]. In our perspective, *R. faecalis* PA2 cultured under the aforementioned conditions could be an alternative source for microbial-based functional ingredients. Although *R. faecalis* PA2 could provide several beneficial metabolites and it is a promising source for alternative microbial-based functional ingredient, further investigation *in vivo* is required to verify its safety and efficiency before practical application.

## Author contribution statement

Chewapat Saejung: Conceived and designed the experiments; Performed the experiments; Analyzed and interpreted the data; Contributed reagents, materials and analysis tools; Wrote the paper.Khomsorn Lomthaisong: Contributed reagents and analysis tools. Prawphan Kotthale: Performed the experiments; Analyzed and interpreted the data.

## Data availability statement

Data will be made available on request.

## Declaration of competing interest

The authors declare that they have no known competing financial interests or personal relationships that could have appeared to influence the work reported in this paper.
