# Supplementary material for: Alternative microbial-based functional ingredient source for lycopene, beta-carotene, and polyunsaturated fatty acids
Source: Heliyon. 2023 Feb 17;9(3):e13828. doi: 10.1016/j.heliyon.2023.e13828 (PMC9981927; doi:10.1016/j.heliyon.2023.e13828)
Supplement: Multimedia component 1 [file mmc1.pdf]

Table S1 The kinetic characteristics of *Rhodopseudomonas faecalis* PA2 cultivated in the optimal conditions in a 5-L photo-bioreactor.

**Kinetic characteristics**	**Value**
Maximum specific growth rate (/day)	0.66 ± 0.01
Carotenoid yield (mg/g)	280.28 ± 5.23
Carotenoid productivity (mg/L/day)*	45.37 ± 6.43
Microbial lipid yield (mg/g)	99.91 ± 1.12
Microbial lipid productivity (mg/L/day)*	13.86 ± 0.59

* Calculated from 14 days.
